# Coamorphous Systems of Valsartan: Thermal Analysis Contribution to Evaluate Intermolecular Interactions Effects on the Structural Relaxation

**DOI:** 10.3390/molecules28176240

**Published:** 2023-08-25

**Authors:** Bruno Ekawa, Hermínio P. Diogo, Ricardo A. E. Castro, Flávio J. Caires, M. Ermelinda S. Eusébio

**Affiliations:** 1 Institute of Chemistry, São Paulo State University (UNESP), Araraquara 14801-970, Brazil; bruno.ekawa@unesp.br; 2 Coimbra Chemistry Center, Institute of Molecular Sciences, Department of Chemistry, University of Coimbra, 3004-535 Coimbra, Portugal; rcastro@ff.uc.pt; 3 Centro de Química Estrutural, Institute of Molecular Sciences, Departamento de Engenharia Química, Instituto Superior Técnico, Universidade de Lisboa, 1049-001 Lisboa, Portugal; hdiogo@tecnico.ulisboa.pt; 4 School of Sciences, São Paulo State University (UNESP), Bauru 17033-360, Brazil

**Keywords:** valsartan, coamorphous, glass transition, isoconversional kinetics, TSDC

## Abstract

Coamorphous formation in binary systems of valsartan (Val) with 4,4′-bipyridine (Bipy) and trimethoprim (Tri) was investigated for mixtures with a mole fraction of 0.16~0.86 of valsartan and evaluated in terms of the glass transition temperature. The glass transition of the systems had a behavior outside the values predicted by the Gordon–Taylor equation, showing that Val-Bipy (hydrogen bonding between the components) had a lower deviation and Val-Tri (ionic bonding between the components) had a higher deviation. Mixtures of compositions 2:1 Val-Bipy and 1:1 Val-Tri were selected for further investigation and verified to be stable, as no crystallization was observed during subsequent heating and cooling programs. For these systems, the effective activation energy during glass transition was evaluated. Compared to pure valsartan, the system with the lower glass transition temperature (Val-Bipy) presented the highest effective activation energy, and the system with the higher glass transition temperature (Val-Tri) presented a lower effective activation energy. The results presented a good correlation between the data obtained from two different techniques to determine the fragility and effective activation energy: non-isothermal kinetic analysis by DSC and TSDC.

## 1. Introduction

Valsartan (Val), an antagonist of angiotensin II, is applied as an antihypertensive drug [1]. However, the drug has low aqueous solubility, being categorized as Class II in the Biopharmaceutical Classification System (BCS). Approaches to increase the solubility of valsartan have been made, culminating in the synthesis of the well-known cocrystal system of valsartan:sacubitril [2].

Nowadays, other approaches to increase the aqueous solubility of valsartan have been proposed, one of them being the formation of coamorphous systems [3,4,5]. Coamorphous systems are homogeneous non-crystalline phases made up of two or more low-molecular-weight components. They have the advantage of increased solubility when compared to crystalline phases, due to a higher chemical potential of the components, showing a decrease in hygroscopicity and higher physical stability when compared to a single component amorphous system, due to intermolecular interactions between the components [6,7,8]. Additionally, a higher drug loading capacity is possible when a comparison is made with traditional amorphous solid dispersions that use polymers [6].

Despite coamorphous phases being expected to have an increased physical stability when compared to pure component amorphous phases, they also tend to crystallize, decreasing the system’s Gibbs energy. Knowledge on properties related to molecular mobility in the coamorphous phase, at the glass transition region, and in the supercooled liquid is therefore of great relevance.

Methods for predicting the formation of coamorphous systems can be found in the literature [8,9], as well as approaches to predict the glass transition temperature as a function of the composition [10,11,12] and of the prediction of relaxation times [13]. A relationship between β-relaxation and physical stability was proposed [14]; however, to the best of our knowledge, an investigation of the activation energy of α-relaxation by differential scanning calorimetry, DSC, has not been applied.

The activation energy determined by isoconversional kinetic methods can provide insights into the Vogel–Fulcher–Tammann (VFT) behavior of the system, also allowing one to determine the fragility (*m*) of supercooled liquid [15,16,17,18].

**Figure 1:**
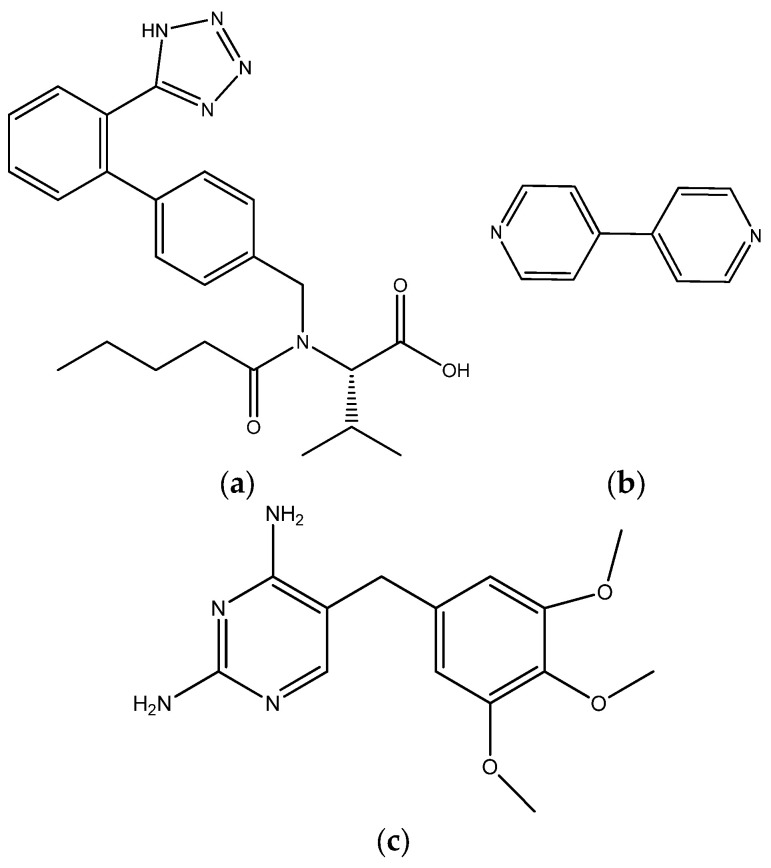
Structural formula of (**a**) valsartan; (**b**) 4,4′-bipyridine; (**c**) trimethoprim.

In this work, the formation of coamorphous binary systems of valsartan (Figure 1a), and the coformers 4,4′-bipyridine (Bipy) (Figure 1b) and trimethoprim (Tri) (Figure 1c) is investigated. These two coformers were selected in order to investigate coamorphous phases stabilized by different intermolecular interactions. Although 4,4′-bipyridine has no pharmaceutical applications, it is a simple model coformer, extensively used in multicomponent solid forms investigation [19,20], as it has a single type of hydrogen-bond acceptor group. Trimethoprim, a dihydrofolate reductase inhibitor, in addition to hydrogen bonding both as a donor and acceptor, may also give rise to salts with valsartan. Hydrogen-bonded coamorphous systems were obtained for Val-Bipy, whereas a coamorphous salt formation was in fact observed for the Val-Tri binary system. For selected compositions of both systems, the results obtained for activation energies of the structural relaxation derived from the Advanced Isoconversional Method–Vyazovkin Method (VM) and also from the Thermally Stimulated Depolarization Current (TSDC) technique, along with the dynamic fragility *m*, were compared and discussed.

## 2. Results and Discussion

The results and discussion will be presented in the following order: (1) the coamorphous systems will be discussed in terms of thermal behavior (DSC), intermolecular interactions (FTIR), and the presence of crystalline material in molar fractions (0.16~0.86); (2) after the preliminary definition of the systems, the glass transition temperature against molar fraction diagrams will be presented and discussed; (3) the systems chosen in the second step will be discussed in terms of the effective activation energy obtained through the advanced isoconversional method of Vyazovkin and also through the Thermally Stimulated Depolarization Current (TSDC) technique.

### 2.1. Coamorphous Systems Characterization

The DSC of the samples obtained after milling may have a small amount of adsorbed water; therefore, tests conducted in closed crucibles and crucibles suitable for volatile substances resulted in glass transitions at different temperatures that could be due to adsorbed water. Therefore, the samples were kept in a desiccator with P_2_O_5_ during 7 days prior to the analysis to decrease the water content present in the samples.

**Figure 2:**
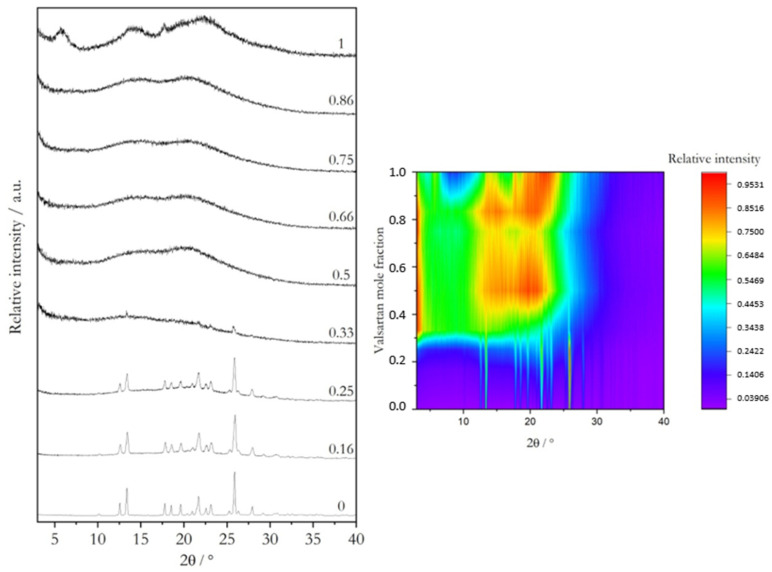
X-ray powder diffractograms of as-received valsartan and 4,4′-bipyridine and of binary mixtures prepared by mechanochemistry (see Section 3.1) with different valsartan mole fractions (indicated in the graph). Projection on valsartan mole fraction—2*θ* plane, color-coded for intensity from dark purple to orange.

**Figure 3:**
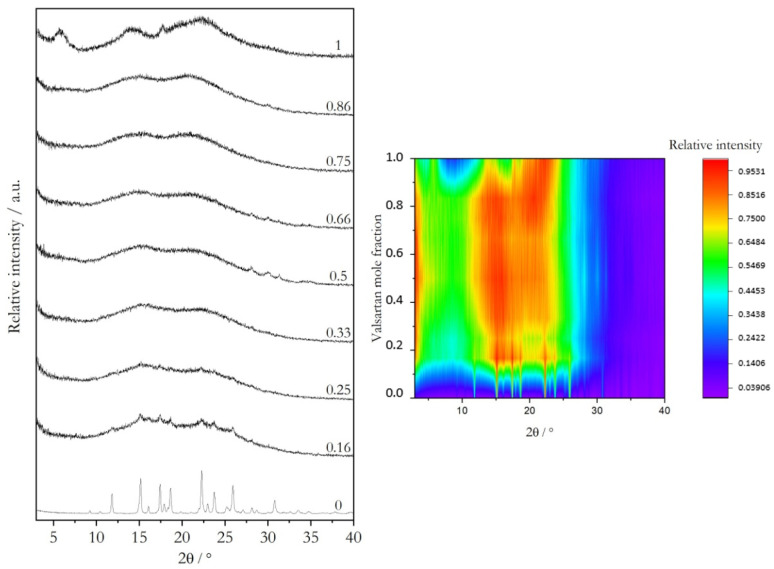
X-ray powder diffractograms of as-received valsartan and trimethoprim and of binary mixtures prepared by mechanochemistry (see Section 3.1) with different valsartan mole fractions (indicated in the graph). Projection on valsartan mole fraction—2*θ* plane, color-coded for intensity from dark purple to orange.

**Figure 4:**
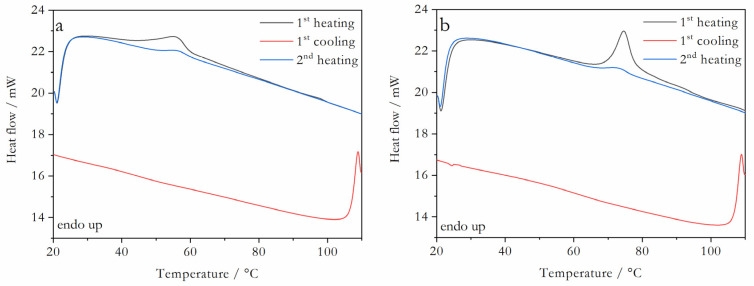
DSC curves of valsartan:4,4′-bipyridine representative mixtures: valsartan mole fraction (**a**) x = 0.5; (**b**) x = 0.66; β = 10 °C min^−^^1^.

**Figure 5:**
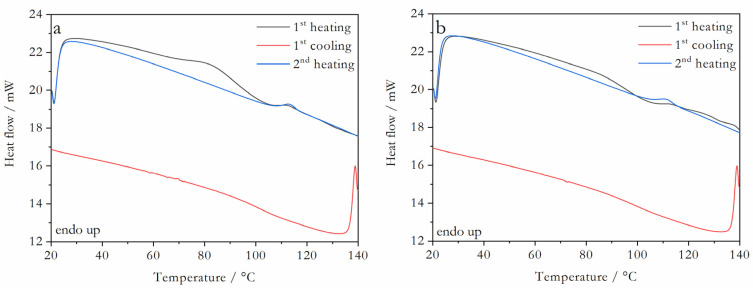
DSC curves of valsartan: trimethoprim representative mixtures: valsartan mole fraction (**a**) x = 0.33; (**b**) x = 0.5; β = 10 °C min^−^^1^.

The X-ray powder diffractograms obtained for Val:Bipy and for Val:Tri mixtures are shown in Figure 2 and Figure 3, respectively, and the representative DSC curves are shown in Figure 4 (valsartan mole fractions x = 0.5 and 0.66) and Figure 5 (valsartan mole fractions x = 0.33 and 0.5). DSC curves for mixtures of other compositions are presented in Figures S1 and S2 of the electronic supplementary material, ESI. It is worth mentioning that according to Mizoguchi et al. [8] and Chambers at al. [9] criteria, these binary systems are predicted to be potential coamorphous formers (Table S1, ESI).

XRPD data confirm a complete amorphization for valsartan compositions from a 0.5 to 0.86 mole fraction in the Val:Bipy system, and from 0.33 to 0.86 for Val:Tri. A single glass transition event is observed in all DSC curves, as expected for a coamorphous system.

**Figure 6:**
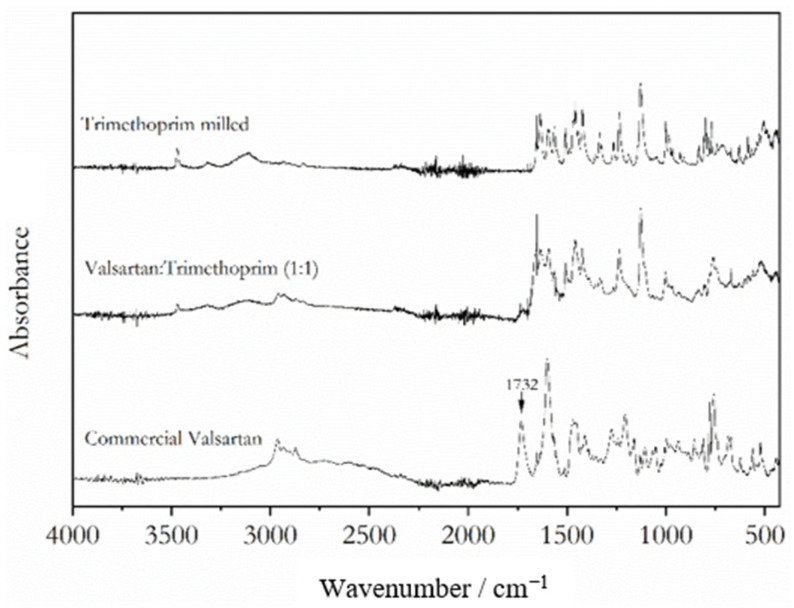
FTIR spectra of valsartan, trimethoprim and valsartan:trimethoprim (1:1) coamorphous mixture.

**Figure 7:**
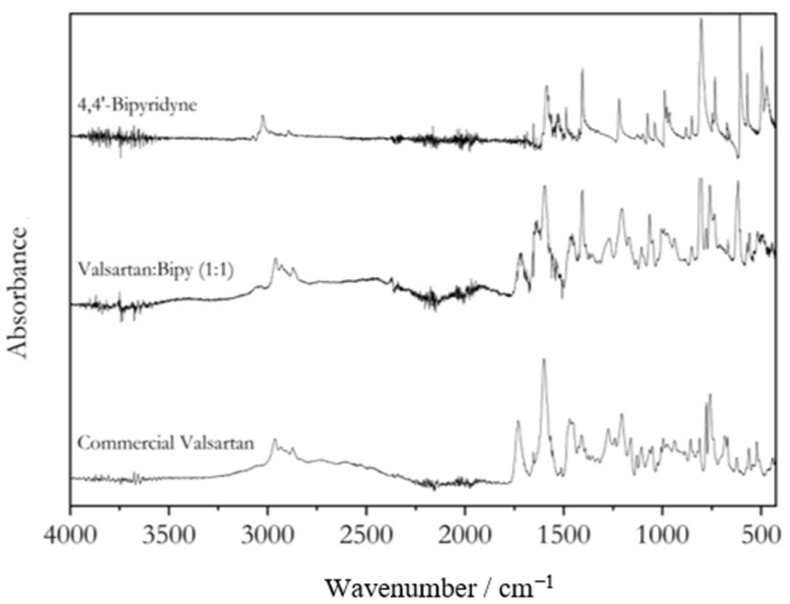
FTIR spectra of valsartan, 4,4′-bipyridine and valsartan: 4,4′-bipyridine (1:1) coamorphous mixture.

FTIR spectra of representative mixtures for each system are shown in Figure 6 and Figure 7. The spectra allow for the characterization of the intermolecular interactions between valsartan and the coformers in these coamorphous systems. The Val:Tri system shows evidence of proton transfer from valsartan to trimethoprim, observed as the disappearance of the valsartan C=O carboxylic acid stretching band (1732 cm^−1^) (Figure 6) and the appearance of the stretching bands ascribed to the symmetric and assymmetric bands of the carboxylate anion at 1506 and 1457 cm^−1^, respectively. The pKa values of valsartan and trimethoprim are 3.9–4.9 [21,22,23] and 7.1 [24], respectively. A difference of base and acid pKa values higher than 3, as observed for this system, is prone to give rise to salts [25,26], which is also observed for this system. As for the Val:Bipy system, two broad bands related to hydrogen bonding between the nitrogen from the pyridine moiety and the carboxylic acid from valsartan appeared in the system, centered at 1920 and 2440 cm^−1^ (Figure 7) [27,28].

After a cycle of heating and cooling of the systems 50 °C above the glass transition, FTIR spectra were measured, and no noticeable changes were observed.

The FTIR spectra of the samples before and after the temperature programs showed that 2:1 Val-Bipy and 1:1 Val-Tri presented: (1) different intermolecular bonds, e.g., ionic (valsartan:trimethoprim) and hydrogen bonding (valsartan:4,4′-bipyridine); and (2) no significant changes in the FTIR spectra and therefore in the interaction between the molecules after the temperature program.

Knowing the interactions in the systems, the glass transition temperature values against the mole fraction of valsartan was compared to the glass transition predicted by the Gordon–Taylor equation [10] in Equation (1):

(1) $$T_{g}=\frac{\omega_{1}T_{g,1}+k\omega_{2}T_{g,2}}{\omega_{1}+k\omega_{2}}$$

where *T_g_* is the glass transition temperature of a mixture with weight fractions ω_1_ and ω_2_ of components 1 and 2, whose glass transition temperatures are *T_g_*_,1_ and *T_g_*_,2_, respectively. *k* is a parameter that can be estimated using the Simha–Boyer equation (*k* = *ρ*_1_*T_g_*_,1_/*ρ*_2_*T_g_*_,2_).

The Gordon–Taylor equation provides a better agreement with mixtures that present an additive behavior, i.e., solid dispersions with less energetic intermolecular interactions (van der Waals). However, behavior outside the Gordon–Taylor equation may provide insights into the interactions [10,11] or may even, when the behavior is not too far from the predicted one, point to a decrease or increase in the free volume.

An addendum should be placed at this point: efforts to amorphize 4,4′-bipyridine and trimethoprim were made by cryo-milling—milling with stainless steel jars cooled in liquid nitrogen before and after 10 min of milling at 30 Hz to a total milling time of 30 min. However, the compounds could not be amorphized by this process. Therefore, the glass transitions for 4,4′-bipyridine and trimethoprim had to be predicted by “2/3 rule” and the linear relationship proposed by Baird [29], respectively. The melting temperatures of 4,4′-bipyridine and trimethoprim, used in these calculations, are 111 °C and 195 °C, respectively, as determined in this work by DSC (Figures S3 and S4, ESI). The *T_g_* values obtained for 4,4′-bipyridine and trimethoprim are −15.7 °C and 59.8 °C, respectively. The *T_g_* value estimated for trimethoprim is in excellent agreement with the experimental value from the literature [30]. For pure valsartan, the experimental glass transition temperature obtained in this work is 70.4 °C (Figure S5), which is in good agreement with values reported in the literature (*T_g_* = 67, 65 [31], *T_g_* = 69 [32]), although a higher value was reported by Xivillé et al. (*T_g_* = 78 °C) [33].

Although glass transition is dependent on the temperature program of both heating and cooling and although the density of the amorphous systems were estimated as 95% [34] of the crystalline ones [35,36], the predicted values could provide insights on how the systems behave compared to the Gordon–Taylor equation.

**Figure 8:**
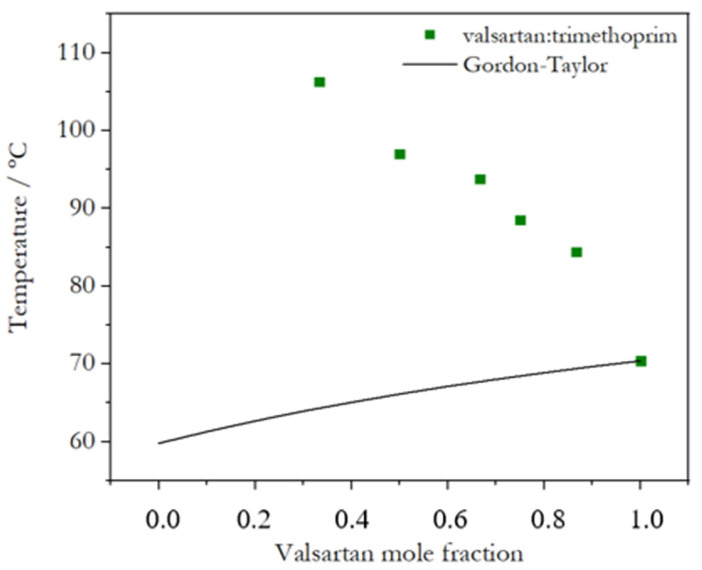
Experimental glass transition temperature of Val:Tri mixtures as a function of valsartan mole fraction; comparison with prediction by the Gordon–Taylor equation.

**Figure 9:**
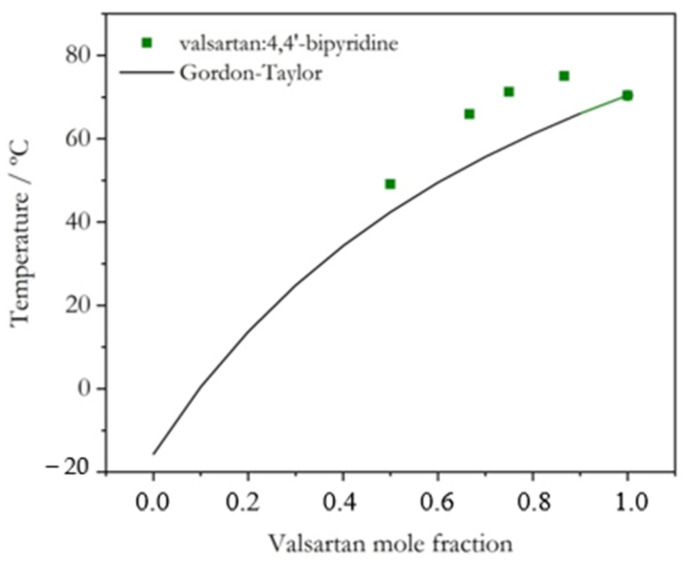
Experimental glass transition temperature of Val:Bipy mixtures as a function of valsartan mole fraction; comparison with prediction by the Gordon–Taylor equation.

The experimental glass transition temperatures as a function of composition are represented in Figure 8 (Val:Tri) and Figure 9 (Val:Bipy), showing that the systems do not follow the Gordon–Taylor equation. Although the results concerning Val:Bipy should be regarded with caution, due to the use of an estimated *T_g_* value for Bipy, this is an expected behavior for systems that are not additive, i.e., systems with charge transfer or hydrogen bonding [11]: there are smaller deviations observed for the hydrogen-bonded (Val-Bipy) binary coamorphous system, while deviations are particularly notorious for the Val:Tri system, as expected for a salt [37].

### 2.2. Activation Energy of Glass Transition for Selected Coamorphous Mixtures

The activation energy of the processes occurring during the glass transition can be calculated by a combination of the Moynihan approach to evaluate the activation energy during glass transition—heating and cooling rates of the same magnitude—and the Advanced Isoconversional Method to evaluate the activation energy by calculating the conversion of the glass to supercooled liquid, α (Equation (2)) [17]:

(2) $$\alpha =\frac{{\left(\Phi -\Phi_{g}\right)|}_{T}}{{\left(\Phi_{l}-\Phi_{g}\right)|}_{T}}$$

where Φ is the heat flow value for the sample, $\Phi_{g}$ is the extrapolated heat flow of the glass, and $\Phi_{l}$ is the extrapolated heat flow of the supercooled liquid at a specific temperature. The activation energy can be calculated following the ICTAC Kinetics Committee methodology, considering small sections of conversion and minimizing the summation in terms of the activation energy, as shown in Equation (3) [18]:

(3) $$\sum_{i}^{n}\sum_{j\neq i}^{n}\frac{J[E_{\alpha },\;T_{i}\left(t\right)]}{J[E_{\alpha },\;T_{j}\left(t\right)]}=n\left(n-1\right)=\Phi (E_{\alpha })$$

where $J\left[E_{\alpha },\;T_{i}\left(t\right)\right]=\int_{a}^{b}exp-\frac{E_{\alpha }}{RT(t)}dt$ can be calculated using the trapezoidal rule.

**Figure 10:**
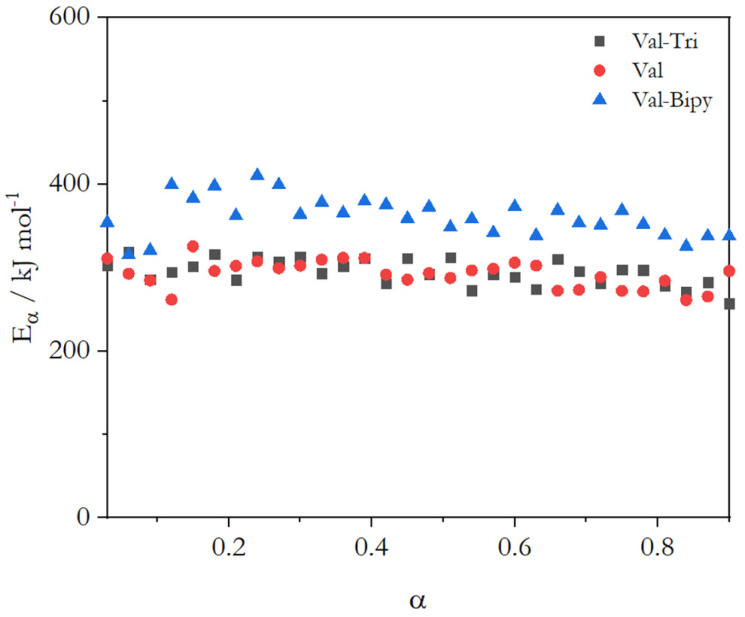
Effective activation energy for the glass transition of valsartan, (2:1) Val-Bipy and (1:1) Val-Tri coamorphous systems.

This methodology was applied to the (2:1) Val-Bipy, (1:1) Val-Tri coamorphous mixtures and also to pure valsartan. The results obtained for pure valsartan (activation energy at glass transition = 309 kJ mol^−1^ and dynamic fragility *m* = 45), calculated using Equation (4) [38], are in good agreement with those obtained by Moura Ramos & Diogo (activation energy 304 kJ mol^−1^ and dynamic fragility *m* = 47) [31]. The effective activation energy of the systems (Figure 10) follows the pattern of glass transition: a decrease in the activation energy with an increasing conversion. However, the energy barrier seems to be higher in the Val-Bipy system, which has a lower glass transition temperature, and an almost similar energy barrier is determined for the Val and Val-Tri systems.

(4) $$m=\frac{E(T_{g})}{2.303RT_{g}}$$

Thermally stimulated depolarization current (TSDC) technique was also used to investigate the dynamics of Val-Bipy (2:1) and Val-Tri (1:1) coamorphous samples. Experimental results, used to access the glass transition temperatures and the activation energies, are presented in Figures S7–S9, in ESI. The glass transition temperature is taken as the maximum temperature value of the highest intensity peak in Figures S8 and S9, for Val-Bipy and Val-Tri, respectively [39].

The temperature-dependent relaxation time, τ(T), associated with a given partial polarization peak, is obtained from *I*(t) raw data by a standard treatment described in detail in [40].

Since the τ(T) lines associated with the alpha mobility modes of motion are generally curved, representing a broad distribution of activation energies of the modes of this relaxation (see insert of Figures S8 and S9, ESI), the calculation of the dynamic fragility of the glass former entity has to be made by non-linear fit. In the present case, the Williams–Landel–Ferry (WLF) equation, which provides the kinetic parameters characterizing the mobility, was used:

Ea (*T_g_*) = 2.303 R × (∂ln*τ*(*T*)/∂(1/*T*)) at (*T* = *T_g_*) (5)

From the Ea (*T_g_*) values obtained by TSDC (see Table 1), the dynamic fragility was calculated also using Equation (4).

Table 1 shows the effective activation energy at *T_g_* and fragility (*m*) obtained by the advanced isoconversional method and TSDC measurements.

Due to the nature of the systems and techniques, a difference in the values obtained from the advanced isoconversional method and TSDC measurement is expected. The first point to mention (and also a hypothesis) is that the presence of charges in the Val-Tri systems gives rise to a different mobility when the system is polarized and that, therefore, the relaxation process differs from the results obtained by DSC. It is worth noting that in the results, the fragility and effective activation energy for the Val-Bipy system were higher than for the other systems (pure valsartan and Val-Tri). This effect may be related to the difference mentioned by Shirai [41], where an increase in fragility magnifies the value of the effective activation energy. In addition, a lower effective activation energy for the Val-Tri system is also supported by the rearrangement of ionic species, as this process tends to lower the energy barrier [42].

However, it is relevant to mention that both techniques (DSC and TSDC) show the same tendency relating to relative fragility for the three studied systems, the most notorious difference existing in the case of the Val-Tri (1:1) system, probably resulting from the ionic bond formed between the two constituents.

## 3. Materials and Methods

### 3.1. Materials

Valsartan was acquired in a local drugstore (Bauru, SP, Brazil, lot 17C-B001-016992). The X-ray powder diffractogram and differential scanning calorimetry heating curve are presented as supplementary material (Figures S5 and S6, ESI) and are in good agreement with the literature [32]. The coformers used in the synthesis of the coamorphous systems were trimethoprim (TCI, x > 98%) and 4,4′-bipyridine (Fluka, x > 99%).

The coamorphous systems were synthesized by neat mechanochemistry in a Retsch MM400 mill. Valsartan:4,4′-bipyridine mixtures were prepared in 10 mL stainless steel jars with two 7 mm stainless steel spheres; the milling time was 30 min at 15 Hz. Valsartan:trimethoprim systems were synthesized in a 10 mL zirconia jar with 16 zirconia spheres (diameter 2 mm); the milling time was 30 min at 30 Hz. Mixtures with valsartan molar fractions of 0.16, 0.25, 0.33, 0.5, 0.66, 0.75, and 0.85 were investigated. It is important to stress that the material of the jars and the frequency used in the experiments were chosen while considering both the highest amorphization capability and the glass transition temperature of the systems. Samples were kept in a dry environment after preparation (desiccator with P_2_O_5_).

### 3.2. Fourier-Transform Infrared Spectroscopy (FTIR)

Spectra were collected in Attenuated Total Reflection (ATR) mode, using a Thermo Nicolet 380 Fourier transform infrared spectrometer (Thermo Scientific TM, West Sacramento, CA, USA), with a Smart Orbit Diamond ATR accessory ((Thermo Scientific TM, West Sacramento, CA, USA). The FTIR spectra were recorded in the region between 4000–400 cm^−1^, with 64 scans and a resolution of 2 cm^−1^.

### 3.3. X-ray Powder Diffraction (XRPD)

X-ray powder diffractograms were obtained using a Rigaku MiniFlex 600 diffractometer (Tokyo, Japan), Cu K_α_ radiation (λ = 1.541862 Å) with a K_β_ radiation filter and a D/teX-Ultra high-speed detector. Silicon was used as external calibrant. Samples were placed in zero background holders that have a 4 mm diameter × 100 μm deep depression in the middle. Spin was used to avoid preferential orientations.

### 3.4. Differential Scanning Calorimetry (DSC)

A Pyris1 PerkinElmer calorimeter (Norwalk, CT, USA) was used, with an intracooler unit at −25 °C (ethylene glycol–water 1:1 *v*/*v* cooling mixture), PerkinElmer 50 μL aluminum crucibles suitable for volatile substances. The analysis was performed with a 20 mL min^–1^ nitrogen purge.

For the determination of the glass transition temperature as a function of composition, sample masses of about 2 mg were used. The temperature program includes a first heating run at 20 °C min^−1^ until 120 °C (Valsartan:4,4′-bipyridine) or 140 °C (Valsartan:Trimethoprim) with a hold of 10 min, followed by a cooling (until 20 °C)/heating cycle at the same rate of |20| °C min^−1^.

For the isoconversional kinetics [15,16,17,18], the chosen samples’ masses were 15 mg to decrease the signal-to-noise ratio for a reliable determination of the conversion. These masses were also used by other authors [16,17]. Heating and cooling rates of β = |5|, |10|, |15|, |17.5|, and |20| °C min^–1^ were employed.

Glass transition temperatures, *T_g_*, were determined as the onset of the step change in heat flow observed at the glass transition. All samples were measured in triplicate.

The temperature of the equipment was calibrated with certified reference materials (indium and phenyl salicylate) for each heating rate, and empty 50 μL aluminum crucibles were used for the baseline correction.

### 3.5. Thermally Stimulated Depolarization Current (TSDS)

Thermally stimulated depolarization current experiments were carried out with a TSC/RMA spectrometer (TherMold, Stamford, CT, USA) covering the range of −50 °C to +120 °C, using liquid nitrogen as a cooling agent. For TSDC measurements, the sample (thickness of ~0.5 mm) was placed between the disc-shaped electrodes (7 mm diameter) of a parallel plate capacitor and immersed in an atmosphere of high-purity helium (1.1 bar).

Global experiments and partial polarization experiments were carried out in the samples, with the polarization being active between *T_p_* (polarization temperature) and *T*_0_ (lower temperature) for the global experiments. In the case of partial polarization experiments, the polarization range consisted of “windows” of *T_p_*-Δ*T*, in which Δ*T* were set at 2 °C.

A detailed description of experimental procedures and calculations concerning thermally stimulated depolarization current technique is available in the works by Diogo et al. [40,43].

## 4. Conclusions

Two coamorphous systems (Val-Bipy and Val-Tri), which were highly stable in order to provide the experimental conditions necessary to measure the effective activation energy of the glass transition, were analyzed. The interaction between valsartan and 4,4′-bipyridine occurred by hydrogen bonding, and the glass transition temperatures of the coamorphous systems with different mole fractions were lower than that of pure valsartan, but also deviated from the glass transition predicted by the Gordon–Taylor equation. The interaction between valsartan and trimethoprim presented an ionic character, and the glass transition temperature was substantially greater than for pure valsartan for some mole fractions (up to 100 °C for a mole fraction of valsartan below 0.5). The effective activation energy of the selected (2:1) Val-Bipy coamorphous system was higher than that of pure valsartan, and for (1:1) Val-Tri, a value close to that of valsartan was obtained. A higher effective activation energy for the Val-Bipy system may be related to a higher fragility and to a lower glass transition temperature, which magnifies the value of the effective activation energy.

## Figures and Tables

**Table 1:** Glass transition temperature, *T_g_*, effective activation energy at *T_g_* and fragility, *m,* for valsartan, (2:1) Val-Bipy and (1:1) Val-Tri coamorphous systems, obtained by advanced isoconversional method and TSDC measurements.

Sample (Stoichiometry)	Glass Transition Temperature (*T_g_*/°C)	Effective Activation Energy at *T_g_* (kJ mol^−1^)	Fragility (*m*)
Valsartan	70 ^a^, 67(DSC) ^b^, 65(TSDC) ^b^	300 ^a^, 328(DSC) ^b^, 304(TSDC) ^b^	46 ^a^, 50(DSC) ^b^, 47(TSDC) ^b^
Val-Bipy (2:1)	62 ^a^, 67 ^c^	363^a^, 376 ^c^	57 ^a^, 58 ^c^
Val-Tri (1:1)	97 ^a^, 96 ^c^	308 ^a^, 369 ^c^	44 ^a^, 49 ^c^

Where: ^a^ results found in the present study from DSC; ^b^ reported by Moura Ramos & Diogo [31]; ^c^ results obtained from TSDC in the present study (see Figure S10 of ESI).

## Data Availability

Not applicable.

## Supplementary Materials

The following supporting information can be downloaded at: https://www.mdpi.com/article/10.3390/molecules28176240/s1. References [8,9] are cited in the supplementary materials.
