# Supplementary material for: Coamorphous Systems of Valsartan: Thermal Analysis Contribution to Evaluate Intermolecular Interactions Effects on the Structural Relaxation
Source: Molecules. 2023 Aug 25;28(17):6240. doi: 10.3390/molecules28176240 (PMC10488875; doi:10.3390/molecules28176240)
Supplement: Supplementary file 1 [file molecules-28-06240-s001.zip › molecules-2525415-supplementary.pdf]

Coamorphous Systems of Valsartan: Thermal Analysis Contribution to Evaluate Intermolecular Interactions Effects on the Structural Relaxation

Bruno Ekawa [1,2], Hermínio P. Diogo [3], Ricardo A. E. Castro [2], Flávio J. Caires [4,*] and M. Ermelinda S. Eusébio [2,*]

[1] Institute of Chemistry, São Paulo State University (UNESP), Araraquara 14801-970, Brazil; bruno.ekawa@unesp.br
[2] Coimbra Chemistry Center, Institute of Molecular Sciences, Department of Chemistry, University of Coimbra, 3004-535 Coimbra, Portugal; rcastro@ff.uc.pt
[3] Centro de Química Estrutural, Institute of Molecular Sciences, Departamento de Engenharia Química, Instituto Superior Técnico, Universidade de Lisboa, 1049-001 Lisboa, Portugal; hdiogo@tecnico.ulisboa.pt
[4] School of Sciences, São Paulo State University (UNESP), Bauru 17033-360, Brazil
* Correspondence: flavio.caires@unesp.br (F.J.C.); quierme@ci.uc.pt (M.E.S.E.)

Table S1 – Valsartan coamorphous formation prediction with the coformers used in this work, 4,4'-bipyridine and trimethoprim, and also with *L*-proline.

Coformer	Mizogochi *et al.* 2019 [1]		Chambers *et al.* 2021 [2]
**Trimethoprim**	$\Delta logP$ = 3,3	ΔH_{mix}=-1,3	COAM *value* = 0.67
**4,4'-bipyridine**	$\Delta logP$ = 3,0	ΔH_{mix}=-2,4	COAM *value* = 0.53
***L*-Proline**	$\Delta logP$ = 6,9	ΔH_{mix}=-0,4	COAM *value* = 0.08

Criteria for coamorphous formation: Mizoguchi: $\Delta logP \leq 6$ and ΔH_{mix} negative; Chambers: COAM *value* close to unity The colors show how adequate the results are following the procedures suggested by the authors. red: higher than the value suggested by Mizoguchi *et al.* or lower than the value suggested by Chambers *et al.* yellow: an intermediate value than the suggested by Chambers *et al.*; green: values in agreement with the proposed by Mizoguchi *et al.* or a value close to the suggested by Chambers *et al.*.

The equation proposed by Chambers *et al.* 2021 [2] for the COAM prediction value.

$$= (-0.123 \times \Delta H_{hb}) + (-0.136 \times \Delta H_{mix}) + (-0.00350 \times \Sigma HBC_{self}) + (0.00297 \times AV.MW) + (-0.00176 \times \Delta TPSA) + (0.0105 \times \Delta\mu) + (-0.0441 \times \Delta(\delta h)) + (-0.204)$$

where, the parameters ΔH_{hb}, hydrogen bonding excess enthalpy; ΔH_{mix} excess enthalpy of mixing, AV. MW, average molecular weight of valsartan and the co-former; ΔTPSA, difference between the topological polar surface area of valsartan and the co-former; $\Delta(\delta d)$, $\Delta(\delta p)$, $\Delta(\delta h)$, difference of the Hansen parameter for dispersion, dipole-dipole interaction, hydrogen bonding between valsartan and the co-former; ΣHBC_{self}, sum of the difference of hydrogen acceptor and donor sites of each component; and $\Delta\mu$, the difference between the pseudo-chemical potential of the pure components. The parameters (ΔH_{hb}, ΔH_{mix}, δd, δp, δh, and $\Delta\mu$) were obtained from Cosmo Quick following the approach used by Chambers *et al* [2].

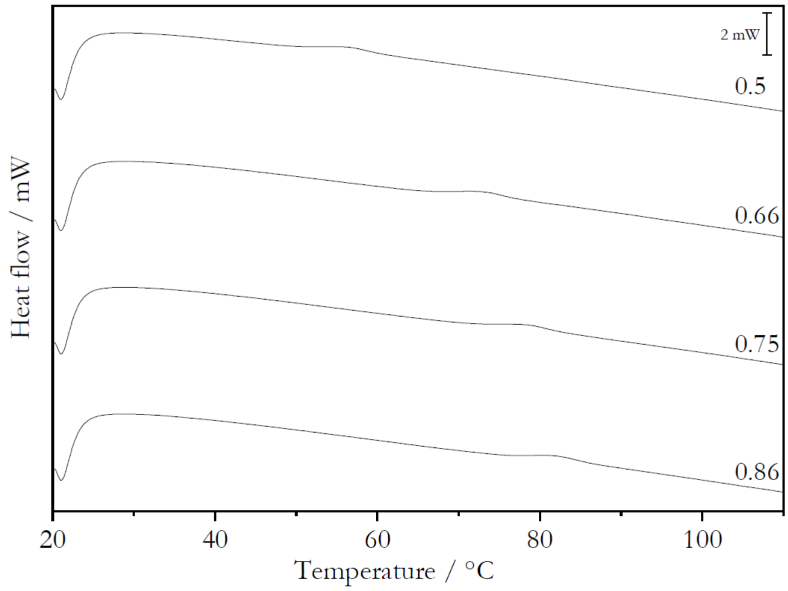

Figure S1 – DSC second heating runs of Val:Bipy mixtures with different valsartan mole fractions, indicated next to the respective curve; β = 10 °C min^{-1}.

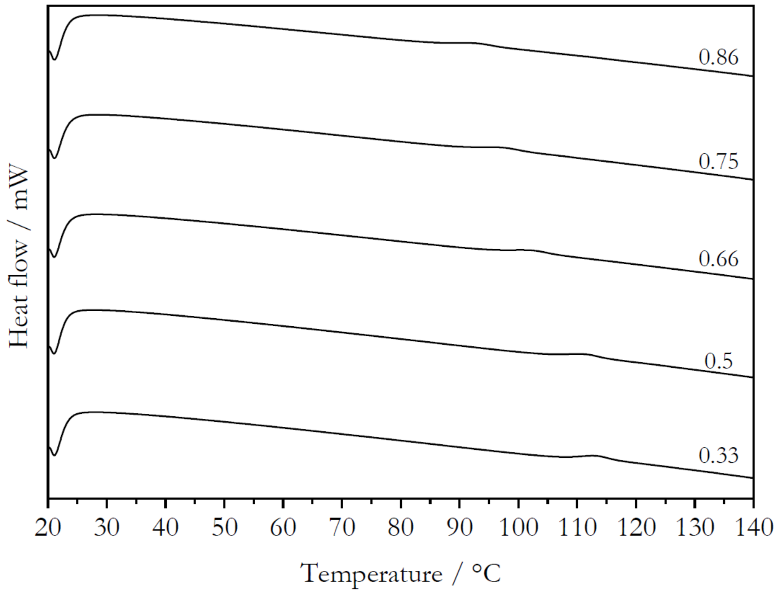

Figure S2 – DSC second heating runs of Val:Tri mixtures with different valsartan mole fractions, indicated next to the respective curve; β = 10 °C min^{-1}.

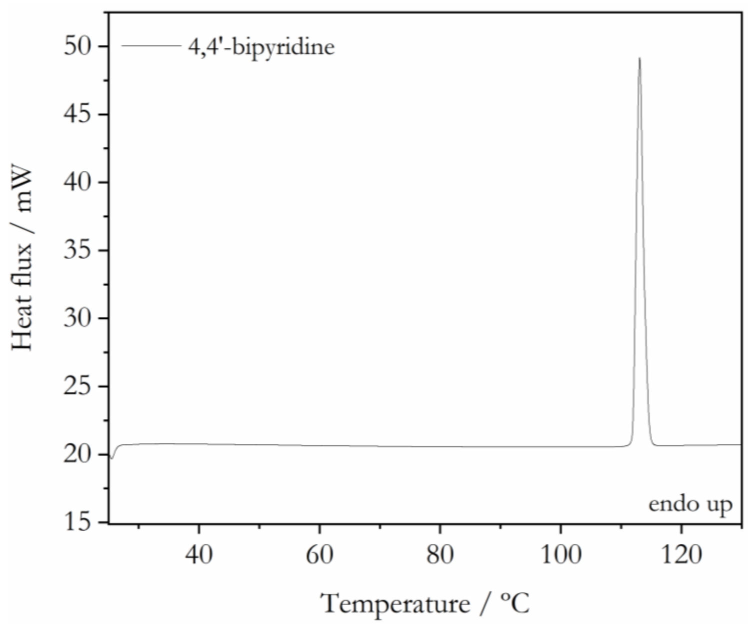

Figure S3 – DSC heating curve of pure 4,4'-bipyridine, β = 10 °C min^{-1}.

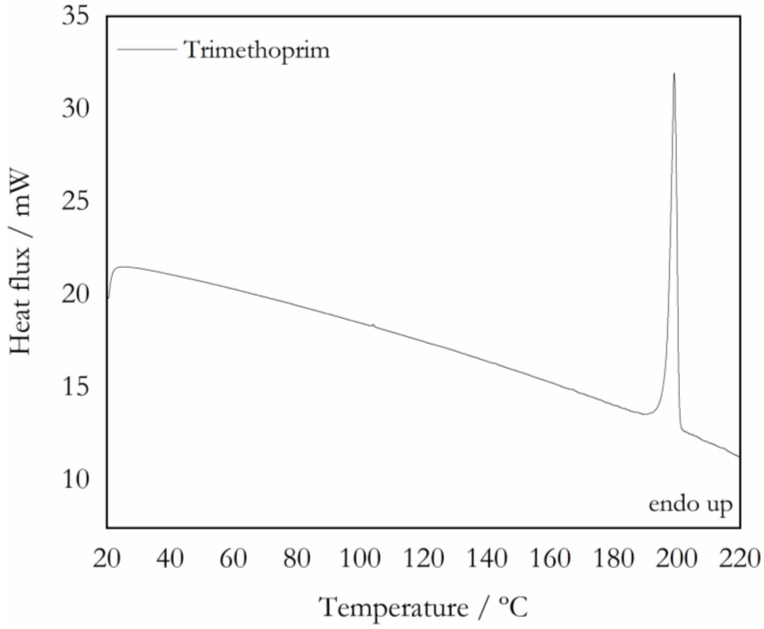

Figure S4 – DSC heating curve of pure trimethoprim, β = 10 °C min^{-1}.

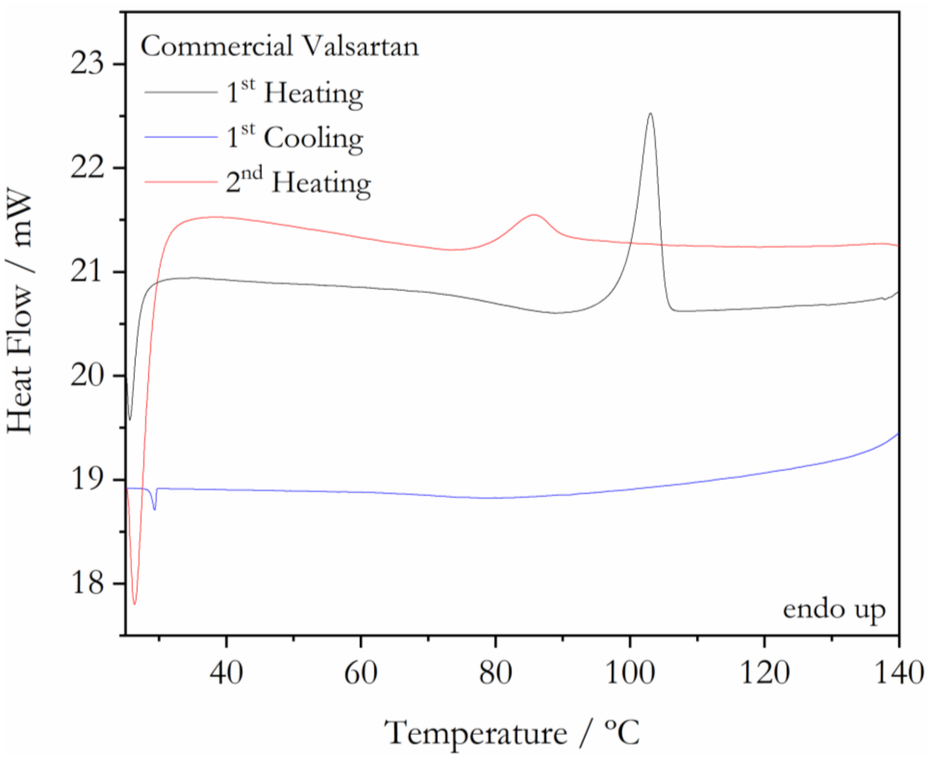

Figure S5 – DSC curves of pure valsartan, β = 10 °C min^{-1}.

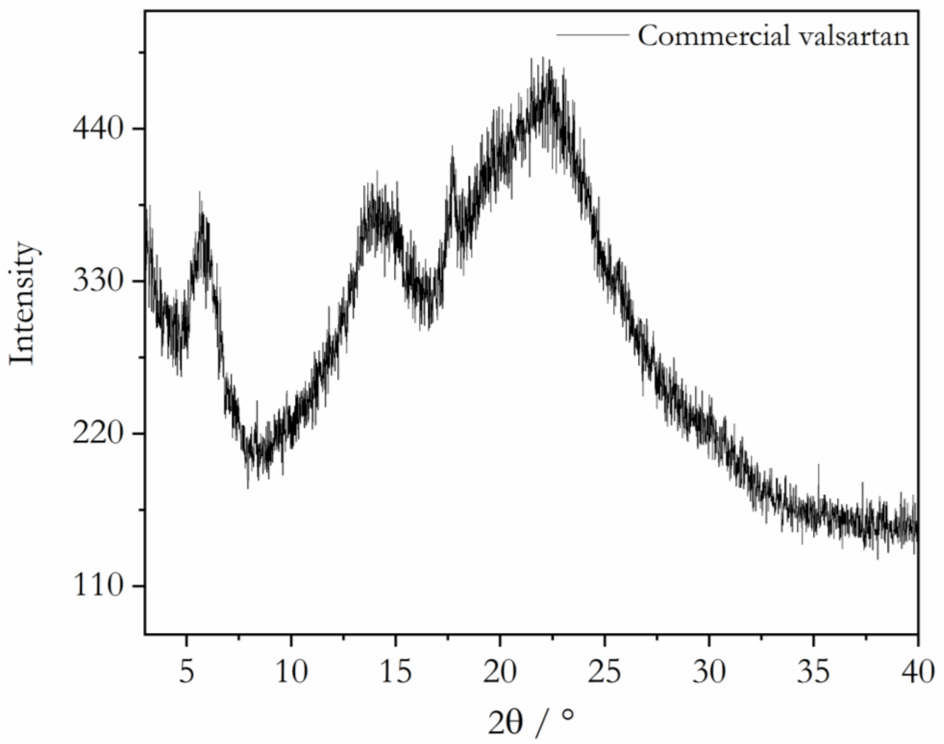

Figure S6 – X-ray powder diffractogram of commercial valsartan.

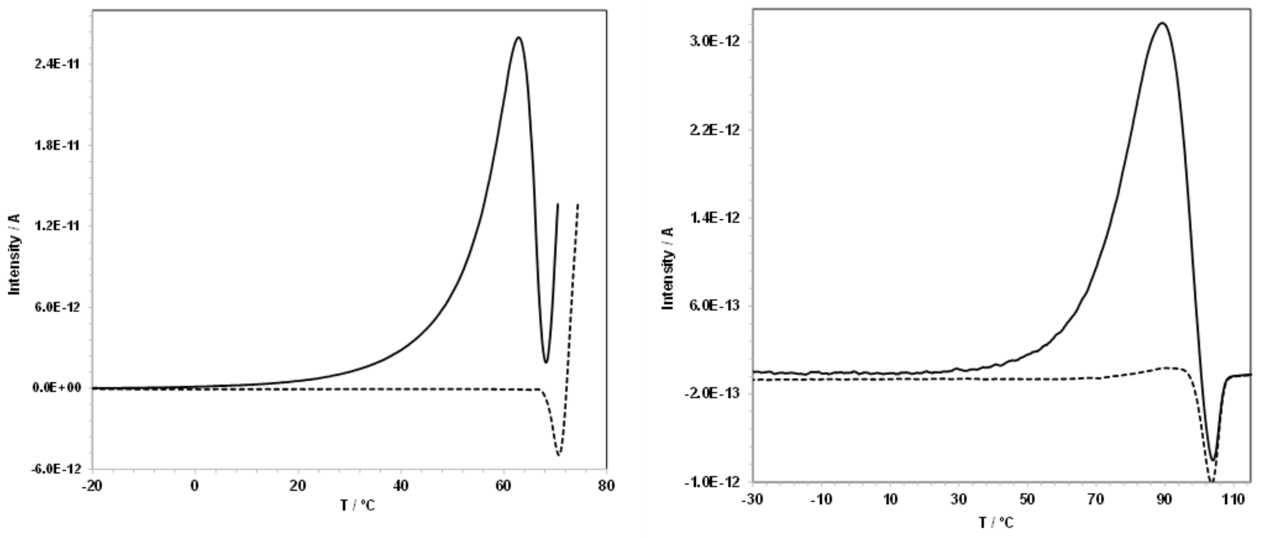

Figure S7 – Structural relaxation of Val-Bipy (left) and Val-Tri (right) observed in a global TSDC experiment mode. Polarization temperature: T_P= 70ºC and T_P= 105ºC for Val-Bipy and Val-Tri, respectively. Other experimental conditions: polarizing electric field strength E_P = 250 V mm^{-1} and heating rate q = 8 K min^{-1}. The dotted line describes a similar experiment in the absence of applied electric field (baseline).

Figures S8 and S9 shows a set of narrow polarization window TSDC peaks corresponding to glass transition motional modes of VAL-BIP and VAL-TRI, respectively. The most intense peak, i.e., the one that present the highest dielectric strength (largest area), has a maximum intensity temperature, T_{max}, and is considered the glass transition temperature provided by the TSDC technique $T_{g(TSDC)}$.

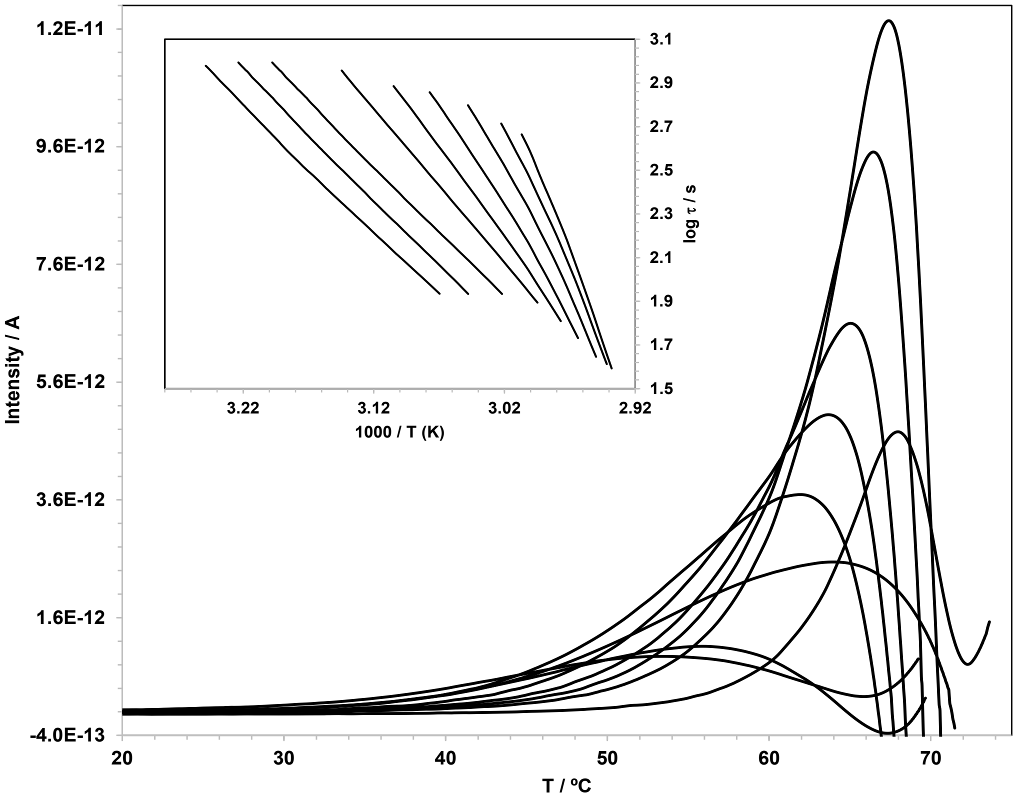

Figure S8 – Motional modes of the structural relaxation of Val-Bipy. The width of the polarization window was two degrees and the polarization temperatures (T_p) ranged from 42 to 66ºC every three degree. Other experimental conditions: polarizing electric field strength E_P = 300 V mm^{-1} and heating rate q = 6 K min^{-1}. The insert shows the τ(T) lines of the motional modes displayed in the main figure.

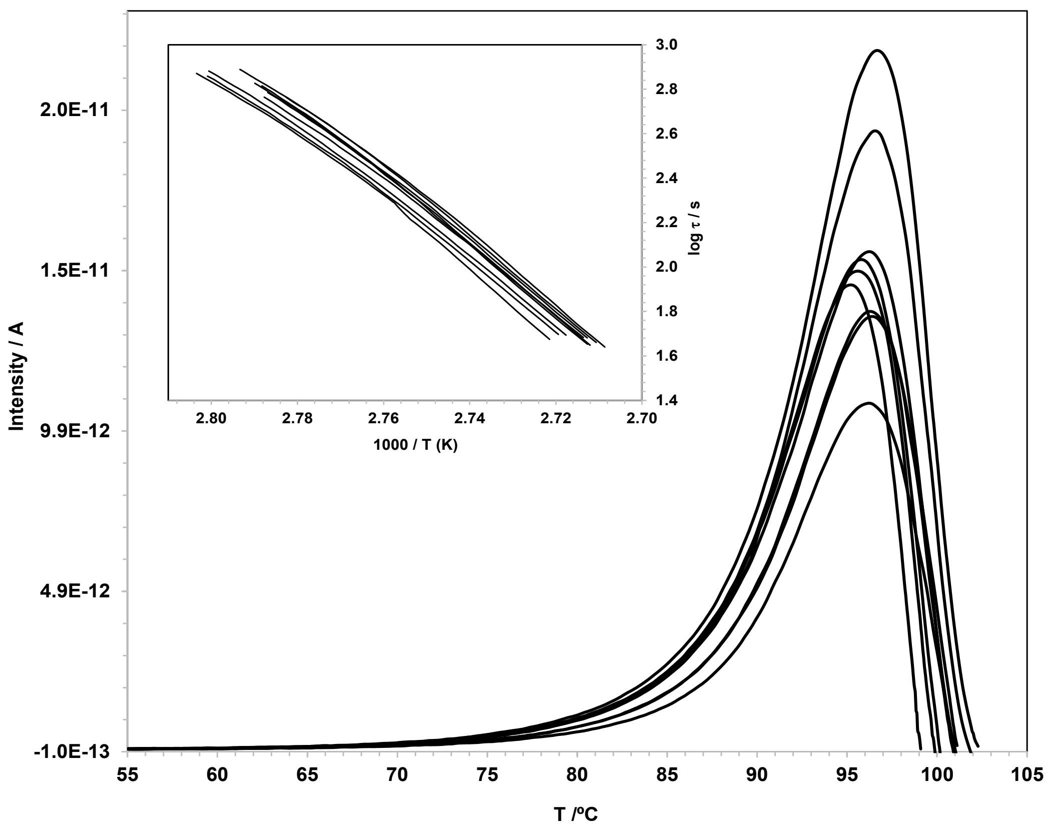

Figure S9 – modes of the structural relaxation of VAL-TRI. The width of the polarization window was two degrees and the polarization temperatures (T_p) ranged from 86 to 94ºC every degree. Other experimental conditions: polarizing electric field strength E_P = 300 V mm^{-1} and heating rate q = 6 K min^{-1}. The insert shows the τ(T) lines of the motional modes displayed in the main figure.

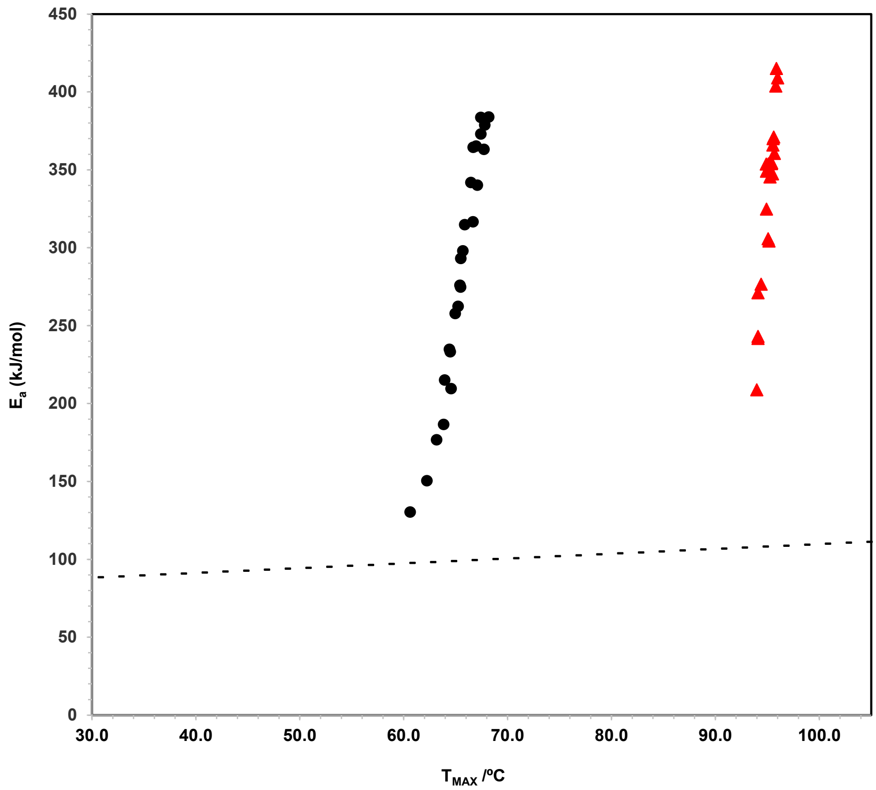

Figure S10 – TSDC relaxation map of the two coamorphous (Val-Bipy and Val-Tri): activation energy, $E_a(T_{Max})$, of the partial polarization components of the different relaxations as a function of the peak's location, T_{Max}. The correspondence between the symbols and the glass-formers is as follows: Val-Tri (triangles, red in online edition) and Val-Bpy (circles, black in online edition). The dotted line describes the zero entropy behavior. The points in the vicinity of T_{gTSDC}, showing an effective and continuous deviation from that line reflecting the cooperative modes of structural relaxation

References

1. Mizoguchi R, Waraya H, Hirakura Y. Application of Co-Amorphous Technology for Improving the Physicochemical Properties of Amorphous Formulations. Molecular Pharmaceutics. American Chemical Society; 2019;16:2142–52.

2. Chambers LI, Grohganz H, Palmelund H, Löbmann K, Rades T, Musa OM, et al. Predictive identification of co-formers in co-amorphous systems. European Journal of Pharmaceutical Sciences. Elsevier B.V.; 2021;157.
